# Theoretical and Experimental Studies of Al-Impurity Effect on the Hydrogenation Behavior of Mg

**DOI:** 10.3390/ma15228126

**Published:** 2022-11-16

**Authors:** Jinzhe Lyu, Roman Elman, Leonid Svyatkin, Viktor Kudiiarov

**Affiliations:** Division for Experimental Physics, School of Nuclear Science & Engineering, National Research Tomsk Polytechnic University, 634050 Tomsk, Russia

**Keywords:** magnesium, aluminum, impurity, magnesium hydride, thin film, hydrogen, first-principle calculations

## Abstract

In this paper, we study the influence of hydrogen concentration on the binding energies in magnesium hydrides. The impact of aluminum atom addition on the hydrogenation behavior of magnesium was theoretically and experimentally defined. Doping Al into the Mg lattice allows the uniform hydrogen distribution in both the fcc and bcc Mg lattice at a low hydrogen concentration (H:Mg < 0.875) to be more energetically favorable. In addition, this leads to bcc Mg lattice formation with a uniform hydrogen distribution, which is more energetically favorable than the fcc Mg lattice when the atomic ratio H:Mg is near 0.875. In addition, compared with the pure Mg, in the Al-doped Mg, the phase transition from the hcp to the fcc structure with a uniform distribution of H atoms induces less elastic strain. Thus, the uniform hydrogen distribution is more favorable, leading to faster hydrogen absorption. Pure magnesium is characterized by cluster-like hydrogen distribution, which decreases the hydrogen diffusion rate. This leads to the accumulation of a higher hydrogen concentration in magnesium with aluminum compared with pure magnesium under the same hydrogenation regimes, which is confirmed experimentally.

## 1. Introduction

Hydrogen is used in various fields of science and technology [1,2]. To achieve the effective development and application of hydrogen energy, especially in the growing market for mobile devices and unmanned aerial vehicles, both of which require small-sized energy sources based on fuel cells, the three problems of clean hydrogen production, compact storage, and efficient transportation need to be solved. Hydrogen storage is an important unsolved problem in the practical application of hydrogen energy [3,4,5]. Metal hydrides can be considered one of the most promising, safe, and efficient ways to store hydrogen. Modern hydride-forming materials capable of accumulating a significant amount of hydrogen include various systems based on rare earth metals, titanium-based alloys, zirconium, magnesium, etc. Magnesium hydride, as a hydrogen-storage material, has attracted great attention due to its light weight, high theoretical hydrogen capacity (about 7.6 wt %), and the natural abundance of Mg [6,7,8,9]. However, bulk MgH_2_ is characterized by impractically high temperatures of hydrogenation/dehydrogenation and poor kinetics [10,11]. Thus, the addition of catalytic materials is widely used to improve MgH_2_ properties and improve the performance of hydrogenation/dehydrogenation processes [12]. For these purposes, transition metals or carbon additives are most often considered by researchers [13,14,15]. Additionally, there are many works devoted to other materials and elements that can be used as a dopant for magnesium hydride or as a precursor to improve hydrogen storage properties [16,17,18]. Aluminum is one of the dopants whose effectiveness when added to magnesium has been experimentally confirmed [19,20,21]. In addition to the direct use of metallic Al as a dopant or catalyst to improve the hydrogen storage properties of Mg, complex hydrides can be used as a source for Al [22,23,24]. The results indicating the positive effect of aluminum-doped magnesium require further explanation, which necessitates theoretical calculations and justifications. The combination of experimental and theoretical data will make it possible to clarify and understand the underlying mechanisms and patterns in the interaction of hydrogen with hydride-forming metals. This work focused on the justification of the catalytic effect of aluminum when added to magnesium using an experimental approach and first-principles calculations.

Previous studies [21,25] showed that compared with face-centered cubic (fcc) and hexagonal close packing (hcp) lattices, a body-centered cubic (bcc) lattice has lower packing density. Therefore, the bcc structure has three times as many tetrahedral and octahedral interstitial sites for hydrogen occupancy as in fcc or hcp, which means in a bcc lattice, more hydrogen atoms are expected to be absorbed [26,27,28,29,30]. In addition, non-close-packed bcc structures, which prevent the so-called blocking layer effect [31], give rise to the rate of hydrogen diffusion in bcc lattices being usually several orders of magnitude higher than that of fcc and hcp ones [30,32,33]. Furthermore, DFT calculations show that even though hydrogen with a high concentration (MgH_0.5_) uniformly distributes over both the fcc and bcc Mg lattices, the fcc structure exhibits a lower diffusion coefficient than the bcc structure [31]. It is thought that a bcc-metal layer in contact with Mg layers can stabilize the bcc structure of magnesium as a result of the migration of Mg atoms along the bcc-metal/Mg interface into the bcc-metal layers [31]. However, the effect of doping metal into the Mg lattice on the structure formation during hydrogenation of Mg in a wide range of hydrogen concentrations has yet not been reported. In this regard, the main idea in this study was to reveal the role of aluminum atom addition on the hydrogenation behavior of magnesium using an experimental approach and first-principles studies.

## 2. Materials and Experimental Methods

Coatings of Mg and Mg–10%Al deposited by magnetron sputtering with a direct current source were used as samples to confirm self-consistent calculations. The coatings were hydrogenated from the gas phase using an automated complex Gas Reaction Controller (Advanced Material Corporation, Pittsburgh, PA, USA) at temperatures of 300–330 °C, a pressure of 30 bar, and a hydrogenation time of 300 min. A S-3400N scanning electron microscope (Hitachi, Tokyo, Japan) was used to analyze the microstructure. The hydrogen concentration in the coatings was determined using an RHEN-602 hydrogen analyzer (LECO Corporation, St. Joseph, CA, USA).

## 3. Ab Initio Methods

**Figure 1:**
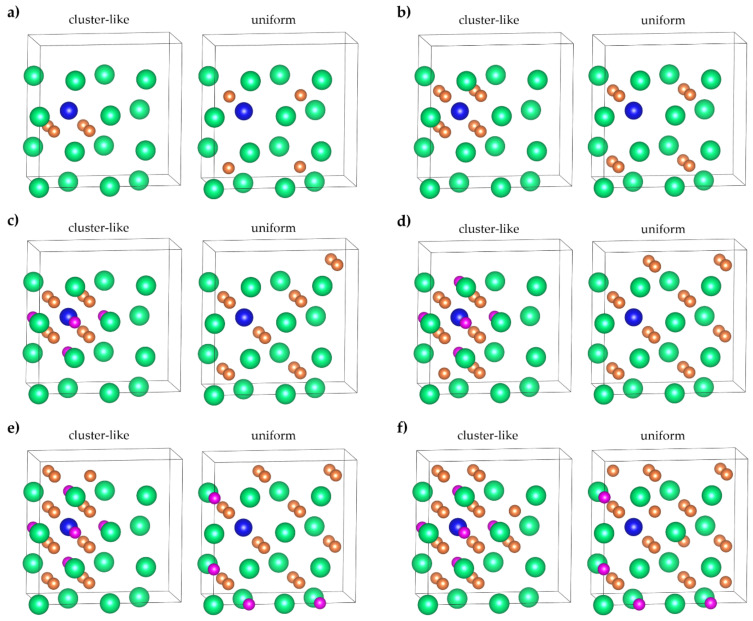
Supercells for fcc (**a**) Mg_15−*x*_Al*_x_*H_4_, (**b**) Mg_15−*x*_Al*_x_*H_8_, (**c**) Mg_15−*x*_Al*_x_*H_12_, (**d**) Mg_15−*x*_Al*_x_*H_16_, (**e**) Mg_15−*x*_Al*_x_*H_20_, and (**f**) Mg_15−*x*_Al*_x_*H_24_. *x* = 0, 1. Left column—H atoms cluster near the Al atom, right column—uniform distribution of H atoms over the supercell. Magnesium atoms are green, aluminum atoms are blue, tetrahedral sites are orange, and octahedral sites are pink.

**Figure 2:**
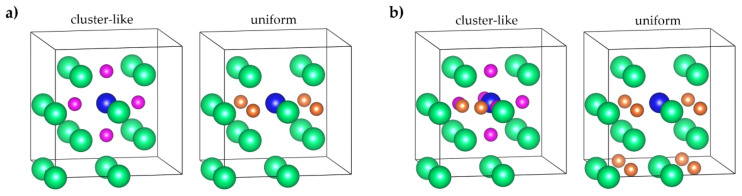

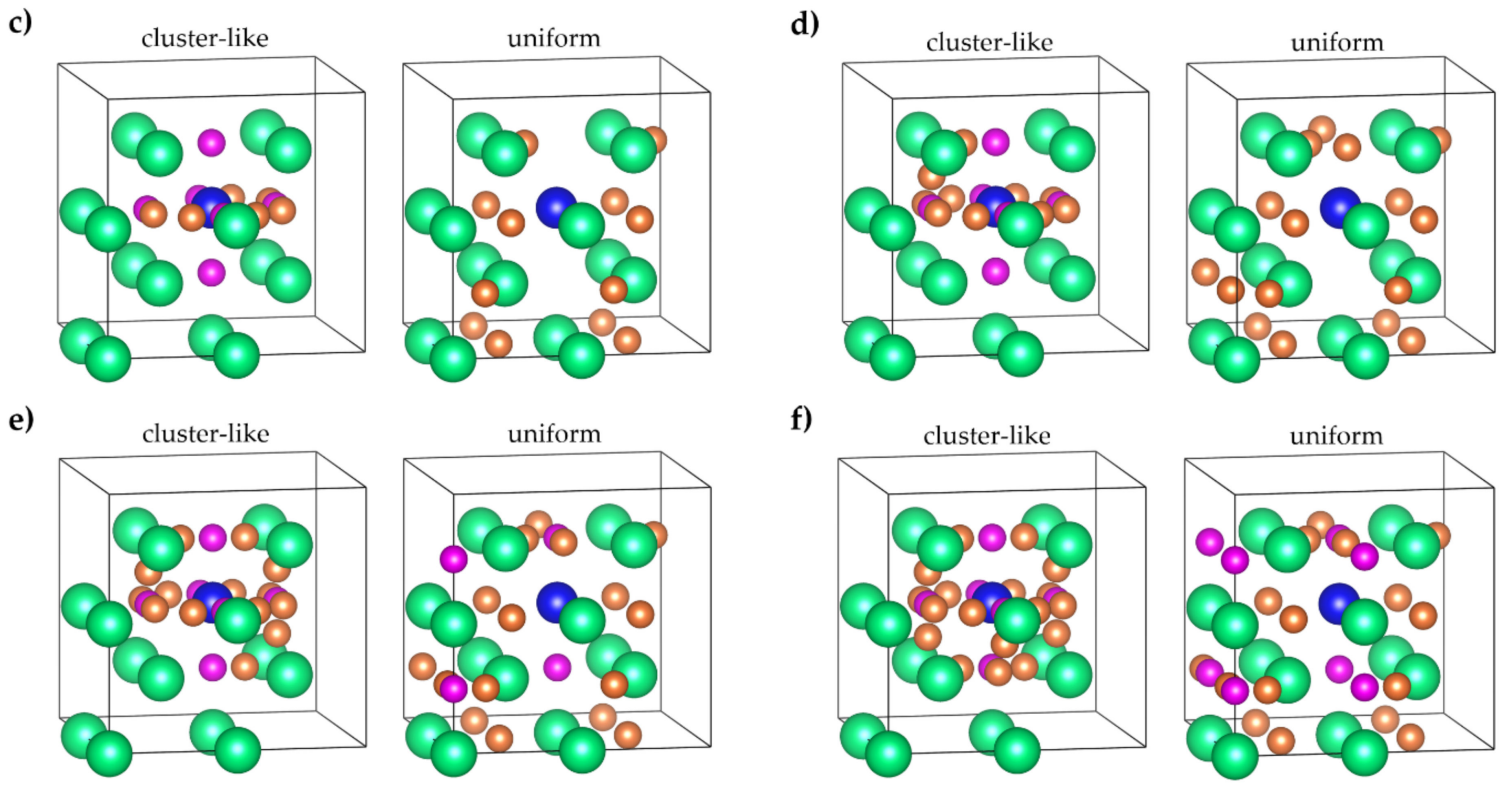
Supercells for bcc (**a**) Mg_15−*x*_Al*_x_*H_4_, (**b**) Mg_15−*x*_Al*_x_*H_8_, (**c**) Mg_15−*x*_Al*_x_*H_12_, (**d**) Mg_15−*x*_Al*_x_*H_16_, (**e**) Mg_15−*x*_Al*_x_*H_20_, and (**f**) Mg_15−*x*_Al*_x_*H_24_. *x*= 0, 1. Left column—H atoms cluster near the Al atom, right column—uniform distribution of H atoms over the supercell. Magnesium atoms are green, aluminum atoms are blue, tetrahedral sites are orange, and octahedral sites are pink.

The ab initio methods for self-consistent calculations were conducted using the open-source program package ABINIT (The ABINIT Group, Belgium). Self-consistent calculations of the total energy of an hcp Mg_16−*x*_Al*_x_* (*x* = 0, 1), an fcc Mg_16−*x*_Al*_x_*H*_y_* (*x* = 0, 1, *y* = 4, 8, 12, 16, 20, 24), and a bcc Mg_16−*x*_Al*_x_*H*_y_* (*x* = 0, 1, *y* = 4, 8, 12, 16, 20, 24) were carried out within density functional theory using the optimized norm-conserving Vanderbilt pseudopotential method [34], as implemented in ABINIT code [35,36]. The exchange and correlation effects were described within the generalized gradient approximation in the form of Perdew–Burke–Ernzerhof (PBE) [37]. The cutoff energy for the plane-wave basis was set to 816 eV. The *k*-point meshes in the structural optimization were set to 5 × 5 × 3 for the hcp Mg_16−*x*_Al*_x_* supercell (*x* = 0, 1), 4 × 8 × 4 for the fcc Mg_16−*x*_Al*_x_*H*_y_* supercell (*x* = 0, 1, *y* = 4, 8, 12, 16, 20, 24), and 6 × 6 × 6 for the bcc Mg_16−*x*_Al*_x_*H*_y_* supercell (*x* = 0, 1, *y* = 4, 8, 12, 16, 20, 24). The hcp Mg_16−*x*_Al*_x_* model was built with Al in the substitution sites consisting of 2 × 2 × 2 hcp Mg unit cells. The fcc Mg_16−*x*_Al*_x_*H*_y_* model was built with Al in the substitution sites and hydrogen in the tetrahedral (T) or octahedral (O) interstitial sites of a supercell consisting of 2 × 1 × 2 fcc Mg unit cells. The bcc Mg_16−*x*_Al*_x_*H*_y_* model was built with Al in the substitution sites and hydrogen in the tetrahedral (T) or octahedral (O) interstitial sites of a supercell consisting of 2 × 2 × 2 bcc Mg unit cells. For a more convenient discussion of the results, the T and O sites in Figure 1 and Figure 2 are enumerated. The relaxation of the atom positions was carried out. The atoms in the system were assumed to be in the equilibrium configuration when the force on each atom was below 5 meV/Å. For the calculation of the fcc and bcc lattice constants, only the optimization of volume was carried out, with a homogeneous dilatation of the three lattice constants. For the calculation of the lattice constants of hcp Mg_16−*x*_Al*_x_* (*x* = 0, 1) supercells, a full optimization of cell geometry was carried out.

(1) $$E_{H}^{b}=\frac{E_{\mathrm{tot}}\left({\mathrm{Mg}}_{16-x}{\mathrm{Al}}_{x}\right)+\frac{y}{2}E_{\mathrm{tot}}\left(H_{2}\right)-E_{\mathrm{tot}}\left({\mathrm{Mg}}_{16-x}{\mathrm{Al}}_{x}H_{y}\right)}{y}$$

Here, *E*_tot_(Mg_16−*x*_Al*_x_*) is the total energy of pure hcp Mg or the hcp Mg_15_Al solid solution supercell because, according to our calculation results, *E*_tot_(hcp Mg_16_) < *E*_tot_(fcc Mg_16_) < *E*_tot_ (bcc Mg_16_), which is in good agreement with the results of theoretical research [38]; *E*_tot_ (H_2_) is the total energy of the hydrogen molecule; *E*_tot_(Mg_16−*x*_Al*_x_*H*_y_*) is the total energy of the fcc or bcc hydride phase, formed during the hydrogenation of pure hcp Mg or the hcp Mg_15_Al solid solution supercell; and *x* and *y* are the numbers of Al and H atoms, respectively, in the Mg-H and Mg-Al-H supercells (*x* = 0, 1; *y* = 4, 8, 12, 16, 20, 24). The calculated hydrogen binding energy in the hydride is energy required to decompose the hydride into pure hcp Mg or the hcp Mg_15_Al solid solution and hydrogen molecules, and it characterizes the energy stability of the compound.

In this work, we did not consider the hcp lattice of Mg_16_H*_y_* and the rutile-like lattice of Mg_16_H*_y_*, because according to calculations in [38], it can be confirmed that: (1) MgH*_x_* with an hcp structure exists in a very low and narrow hydrogen concentration range, so that at the very beginning of the formation of MgH*_x_* (*x* ≈ 0.1), an hcp→fcc phase transformation should occur. (2) The rutile-like lattice of Mg_16_H*_y_* should be formed only when the atomic ratio H:Mg is larger than 1.5. In both fcc and bcc matrixes, the hydrogen diffusion coefficient decreases with hydrogen content (part of the migration paths become unavailable because more and more sites are occupied by hydrogen atoms). For a certain hydrogen content in our considered models, the fastest hydrogen diffusion should be achieved in the case of a uniform distribution of H atoms over the bcc matrix, and the second fastest hydrogen diffusion should be achieved in the case of a uniform distribution of H atoms over the fcc matrix or in the case of a bcc matrix with cluster-like H atoms. Thus, the comparison of the hydrogen diffusion in pure Mg and Al-doped Mg can be replaced by the comparison of the structural stability of pure Mg and Al-doped Mg, both of which show the uniform distribution of H atoms in bcc and fcc matrixes.

## 4. Results and Discussion

**Figure 3:**
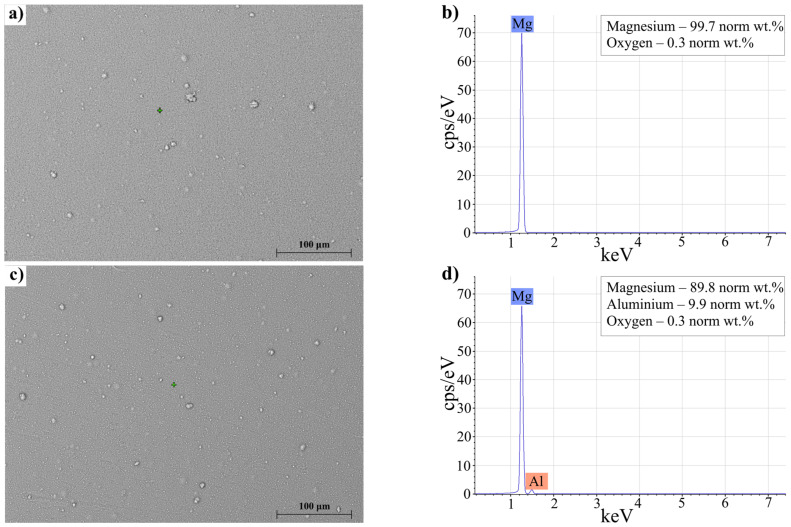
SEM images and EDS spectra of magnesium coating (**a**,**b**) and Mg–10%Al coating (**c**,**d**).

Figure 3 shows scanning electron microphotographs (SEMs) of a Mg coating (Figure 3a) with a spectrum obtained by energy dispersive X-ray spectroscopy (EDS) (Figure 3b), as well as microphotographs of a Mg–10%Al (Figure 3c) with the EDS spectrum (Figure 3d).

The structure of the coatings was homogeneous; microcracks, microvoids, and other visible defects were not observed on the surface. The EDS spectrum of the Mg coating indicated the absence of impurities on its surface. Additionally, according to the EDS spectrum for the Mg–10%Al, the coating consisted of magnesium with aluminum; the aluminum content was 9.9%, which corresponded to the specified ratio.

**Figure 4:**
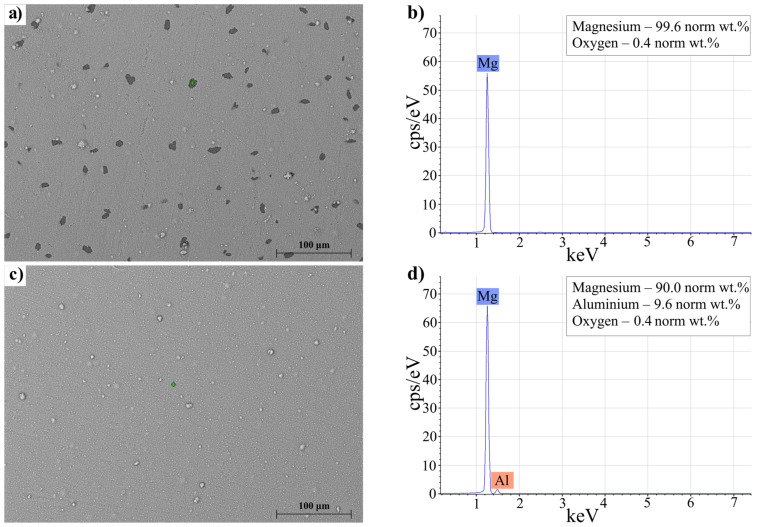
SEM images and EDS spectra of hydrogenated magnesium coating (**a**,**b**) and Mg–10%Al coating (**c**,**d**).

Figure 4 shows SEM images of a hydrogenated Mg coating (Figure 4a) with the EDS spectrum (Figure 4b) and a hydrogenated Mg–10%Al coating (Figure 4c) with the EDS spectrum (Figure 4d). Hydrogenation was performed at a hydrogen pressure of 30 atm, a temperature of 310 °C, and a heating rate of 8 °C per minute.

From the SEM image of the Mg coating (Figure 4a), two specimen regions with different gray levels can be identified. According to the EDS spectrum, the dark areas on the surface, as determined by the BSE mode, consist of pure magnesium and elements that were not captured due to the sensitivity of the energy dispersive X-ray spectrometer to, for example, hydrogen. In this regard, these second-phase precipitates on the surface of the coating represent magnesium hydride [39,40]. The brighter region is metallic Mg, which displays higher secondary electron emission than MgH_2_ [41]. Thus, MgH_2_ nuclei begin to appear on the surface of the magnesium coating during hydrogenation, which grow as magnesium interacts with hydrogen [42]. However, as can be seen from Figure 4c, MgH_2_ nuclei were not visually detected on the surface of the Mg–10%Al coating, which indicated that hydrogen diffused into the bulk of the coating without the formation of the hydride phase on its surface during hydrogenation. This may have indicated a more uniform hydrogen distribution in the coating, whereas for pure magnesium, interaction with hydrogen is accompanied by the formation of hydrides on the surface directly in the process of hydrogenation.

**Figure 5:**
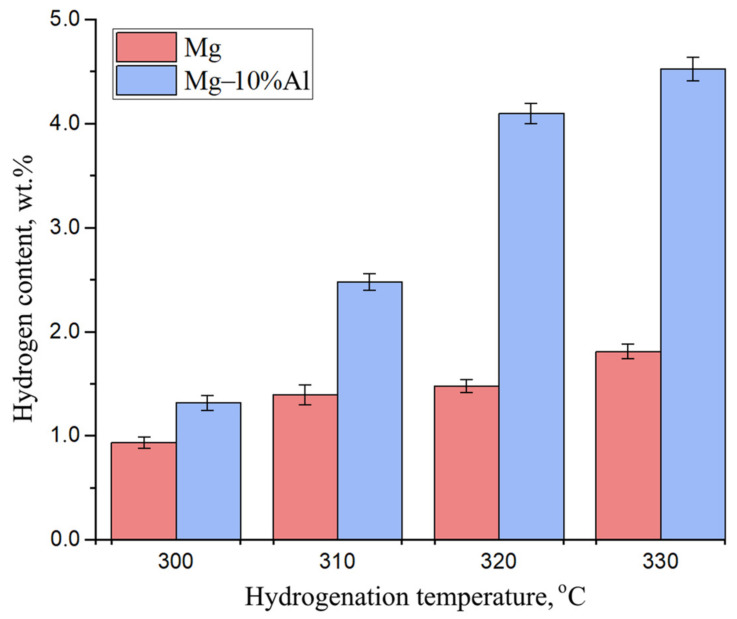
Dependence of hydrogen content versus the hydrogenation temperature in Mg and Mg–10%Al coatings.

Figure 5 represents the hydrogen content in the Mg and Mg–10%Al coatings after hydrogenation at different temperatures. The hydrogen content in each sample was measured at least three times. The relative error was ±2.5%.

As can be seen from the bar chart above, the Mg–10%Al coating had the best hydrogenation rate in the temperature range from 300 to 330 °C, which indicated a catalytic effect of aluminum in Mg. The hydrogen content in the hydrogenated Mg–10%Al coating was more than two times higher than that in the hydrogenated Mg coating after hydrogenation at a temperature of 320 °C.

**Figure 6:**
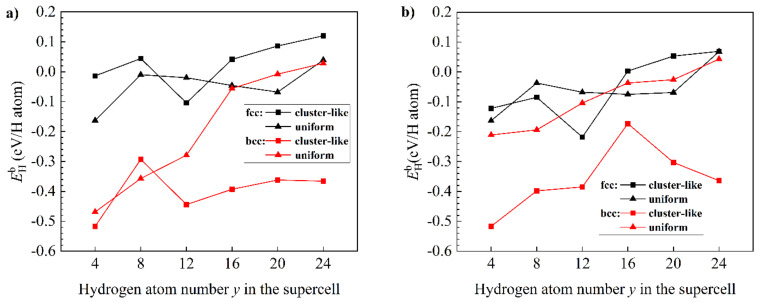
Hydrogen binding energy as a function of hydrogen atom number *y* in the relaxed Mg_16_H*_y_* supercell (**a**), and hydrogen binding energy as a function of hydrogen atom number *y* in the relaxed Mg_15_AlH*_y_* supercell (**b**). For all the structures of Mg_16−*x*_Al*_x_*H*_y_*, two types of hydrogen distribution over the interstitial sites were analyzed versus hydrogenation: cluster-like and uniform distribution.

Self-consistent calculations of the effect of aluminum addition on the structural stability of a solid solution of hydrogen in magnesium and nonstoichiometric magnesium hydrides depending on the hydrogen concentration were carried out to explain the experimentally observed patterns. The calculation results are shown in Figure 6.

From Figure 6a, it can be seen that hydrogen atoms tend to be uniformly distributed over interstitial sites in the bcc-Mg_16_H*_y_* structure, whereas in fcc Mg lattices, hydrogen atoms prefer to occupy adjacent interstitial sites and form clusters, which agrees with the DFT calculation results reported by [38]. Comparing the more stable configurations of hydrogen distribution in the bcc Mg structure, in our calculations (Figure 6a), the stability of the bcc Mg structure increases with the hydrogen concentration. This is in contrast with the calculation results of [38], which show that the enthalpy of formation of the bcc MgH*_x_* structure increases with the hydrogen concentration. We propose that the contradiction arises from the fact that, in [38], the equilibrium cell parameters of all systems were only deduced from the minimization of total energy upon varying both the unit cell volume and the *c*/*a* ratio without considering the relaxation of atomic positions. As seen in Figure 6a,b, doping Al atoms in the Mg lattice increases the stability of the bcc Mg structure with uniform hydrogen distribution, especially when the atomic ratio H:Mg is lower than 0.875, thereby making the bcc Mg structure more stable than the fcc Mg structure when the atomic ratio H:Mg is near 0.875 (Figure 6b). In addition, after doping Al atoms in the fcc Mg lattice, the uniform hydrogen distribution tends to be more stable than the cluster-like hydrogen distribution when the atomic ratio H:Mg is lower than 0.875. Thus, Al-doped hcp Mg can absorb hydrogen faster than pure hcp Mg due to the more favorable uniform hydrogen distribution than the cluster-like hydrogen distribution in the initial hydrogenation stage. Moreover, the addition of aluminum atoms to magnesium with a low hydrogen content leads to a phase transition from the fcc structure to a bcc one, which has a higher hydrogen diffusion coefficient [31].

In addition to the hydrogen binding energy, the elastic strain induced by the volume expansion during hydrogenation can affect the phase transformation. The calculated lattice constants for hcp Mg are *a* = 3.198 Å and *c* = 5.202 Å; the calculated lattice constants for hcp Mg_15_Al are *a* = 3.201 Å and *c* = 5.144 Å. The calculated Mg_16_ and Mg_15_Al supercell volumes are 368.594 Å^3^ and 365.168 Å^3^, respectively. The calculated lattice constants of fcc and bcc Mg_16_H*_y_* and Mg_15_AlH*_y_* (*y* = 4, 8, 12, 16, 20, 24) are presented in Table 1.

**Figure 7:**
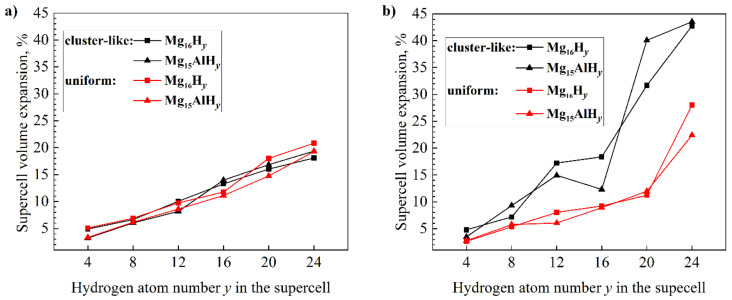
Supercell volume expansion as a function of hydrogen atom number *y* in the relaxed fcc Mg_16−*x*_Al*_x_*H*_y_* (**a**) and bcc Mg_16−*x*_Al*_x_*H*_y_* (**b**) (*x* = 0, 1).

According to these results, the supercell volume expansion can be calculated, which is shown in Figure 7. From Figure 7, it can be seen that doping Al into Mg lattice has a greater effect on volume expansion in the transition from the hcp structure to the bcc structure with a H-cluster-like distribution than on volume expansion in the transition from the hcp structure to other considered structures. Figure 7a shows a lower volume expansion in fcc Mg_15_AlH*_y_* than in fcc Mg_16_H*_y_* over the entire considered hydrogen concentration range for a uniform H distribution and, when H:Mg is below 0.875, for a structure with a H-cluster distribution. It benefits the formation of the fcc structure with the uniform distribution of H atoms.

**Figure 8:**
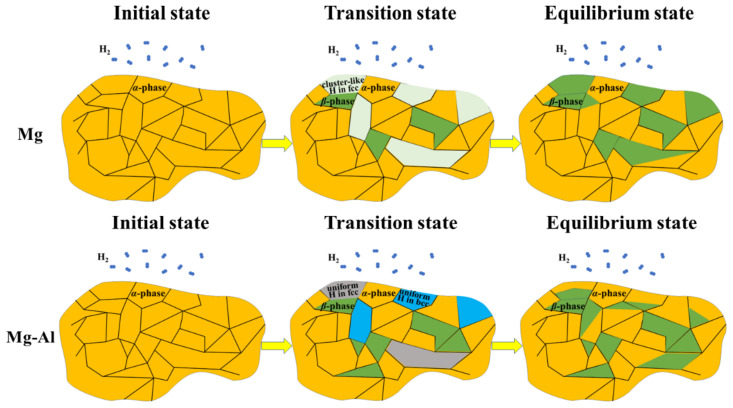
Scheme of hydrogen-induced structural phase transitions in Mg without and with Al additives. The H_2_ molecules, *α* phase, *β* phase, fcc structure with cluster-like H distribution, fcc structure with uniform H distribution, and bcc structure with uniform H distribution are represented, respectively, by dark blue, orange, dark green, light green, grey, and light blue colors.

Finally, on the basis of the present theoretical study reported here as well as experimental data, Figure 8 proposes a scheme of structural hydrogen-induced phase transformations in magnesium without and with Al additives.

In pure Mg, two approaches to the formation of the MgH_2_ hydride (*β*-phase) can be proposed: (1) During hydrogenation of pure Mg due to the continuous diffusion of hydrogen atoms into the interstitial sites of the Mg lattice, a phase transition from the saturated solid solution (*α*-phase) occurs. As hydrogen atoms penetrate the surface and enter the cell of the saturated *α*-phase, an fcc structure with cluster-like H distribution is formed, which leads to a slow diffusion of hydrogen atoms and thereby an accumulation of hydrogen atoms in the structure until the MgH_2_ hydride is formed. (2) The regions with an fcc structure and cluster-like H distribution in Mg bulk can exist for a longer time until the saturation of the hydrogen absorption compared with such regions on the surface. It should be noted that the saturation of the hydrogen absorption is achieved when approximately more than 80% of the powders have surface coverage by hydrides of 80% or more [43]. When the hydrogenation process stops, the H atoms over the regions with an fcc structure and cluster-like H distribution in Mg bulk gather together, with the fcc phase decomposing into the bct *β*-phase and the hcp *α* phase. In Al-doped Mg, in the initial stage of hydrogenation, the bcc and fcc structures with uniform H distribution should be formed, which act as a gateway for fast hydrogen diffusion without the accumulation of hydrogen atoms in the structure. Most of these gateways, especially those on the surface, can exist until the end of the hydrogen absorption and then decompose into the bct *β*-phase and hcp *α* phase.

## 5. Conclusions

Magnesium and its hydrides are of considerable interest for study as hydrogen storage materials. Magnesium has both significant advantages and disadvantages. However, its disadvantages, such as a high hydrogen absorption/desorption temperature, low diffusion rate speed of hydrogen atoms in MgH_2_ layers, and poor kinetics, can be partially compensated by the addition of catalysts, as confirmed by experimental data in many works. In addition to experimental results, it is important to carry out theoretical calculations that substantiate the catalytic effect of various additives. The combination of theoretical calculations and experiments makes it possible to optimize the composition of the magnesium-based material and select the most appropriate hydrogenation parameters.

In our work, the coating of pure magnesium was considered, as well as that of aluminum-doped magnesium. It was demonstrated that hydrogenation at a hydrogen pressure of 30 atm, temperature of 310 °C, and heating rate of 8 °C/min, led to the formation of hydride nuclei on the Mg coating surface. However, under the same hydrogenation conditions, such nuclei could not be observed on the surface of the Mg–10%Al coating, which indicated a more uniform distribution of hydrogen in this coating compared with the hydrogenated Mg coating. Mg–10%Al coatings were characterized by a significantly higher hydrogen content compared with that of the Mg coating. In addition, the hydrogen concentration in Al-doped magnesium increased more strongly with increasing temperature compared with that of pure magnesium. Theoretical calculations based on the ab initio method showed that, compared with the pure Mg, in the Al-doped Mg, when H:Mg was lower than 0.875, although a reduction in the elastic strain from the hcp structure to the fcc structure with the cluster-like distribution of H atoms was observed, the uniform hydrogen distribution was more energetically favorable, which is related to the following factors: (1) For the Al-doped Mg coating, when the atomic ratio H:Mg was lower than 0.875, the uniform hydrogen distribution in both the fcc and bcc structures tended to be energetically more favorable. When the atomic ratio H:Mg was near 0.875, the bcc Mg structure with the uniform hydrogen distribution could be especially more stable than the fcc Mg structure. (2) Over the entire considered hydrogen concentration range, doping Al into the Mg lattice allowed for a reduction in the elastic strain induced by the phase transition from the hcp structure to the fcc structure with the uniform distribution of H atoms. A cluster-like hydrogen distribution was observed in the Mg at the initial stage of hydrogenation. The hydrogenation of pure magnesium was accompanied by the formation of clusters in the coating, which were the centers of attraction and capture hydrogen during its diffusion into the depth of the surface. Subsequently, these clusters formed hydride nuclei, which were visually observed on the surface of magnesium coatings and reduced the rate of hydrogen diffusion. The hydrogenation of the aluminum-doped magnesium coating was characterized by the uniform distribution of hydrogen without the formation of clusters and without decreasing the hydrogen diffusion rate. This leads to the finding that the hydrogen content in the coatings of magnesium with aluminum was greater than in the coatings of pure magnesium after hydrogenation under the same conditions, which was experimentally confirmed.

## Figures and Tables

**Table 1:** The calculated lattice constants of fcc and bcc Mg_16_H*_y_* and Mg_15_AlH*_y_* (*y* = 4, 8, 12, 16, 20, 24) supercells.

System	Configuration	Lattice Constants, Å		System	Configuration	Lattice Constants, Å	
		fcc	bcc			fcc	bcc
Mg_16_H_4_	cluster-like	4.589	3.641	Mg_15_AlH_4_	cluster-like	4.550	3.615
	uniform	4.592	3.616		uniform	4.553	3.606
Mg_16_H_8_	cluster-like	4.615	3.669	Mg_15_AlH_8_	cluster-like	4.592	3.681
	uniform	4.619	3.648		uniform	4.593	3.641
Mg_16_H_12_	cluster-like	4.663	3.780	Mg_15_AlH_12_	cluster-like	4.622	3.743
	uniform	4.659	3.678		uniform	4.628	3.644
Mg_16_H_16_	cluster-like	4.709	3.792	Mg_15_AlH_16_	cluster-like	4.703	3.715
	uniform	4.687	3.692		uniform	4.664	3.677
Mg_16_H_20_	cluster-like	4.746	3.929	Mg_15_AlH_20_	cluster-like	4.742	3.998
	uniform	4.773	3.714		uniform	4.714	3.711
Mg_16_H_24_	cluster-like	4.774	4.037	Mg_15_AlH_24_	cluster-like	4.776	4.031
	uniform	4.811	3.893		uniform	4.775	3.823

## Data Availability

The raw/processed data required to reproduce these findings cannot be shared at this time as the data also forms part of an ongoing study.
